# Reliability and Validity of the Revised Version of the Ability for Basic Movement Scale in Chronic Hospitalized Patients: A Retrospective Study

**DOI:** 10.7759/cureus.89373

**Published:** 2025-08-04

**Authors:** Masatoshi Koumo, Akio Goda, Yoshinori Maki, Kouta Yokoyama, Junichi Katsura, Ken Yanagibashi

**Affiliations:** 1 Rehabilitation, Hikari Hospital, Otsu, JPN; 2 Department of Physical Therapy, Faculty of Health and Medical Sciences, Hokuriku University, Kanazawa, JPN; 3 Neurosurgery, Ayabe Renaiss Hospital, Ayabe, JPN; 4 Neurosurgery, Hikone Chuo Hospital, Hikone, JPN

**Keywords:** ability for basic movement scale, barthel index, chronic condition, functional independence, rehabilitation, reliability, validity

## Abstract

Background and purpose

To investigate the usefulness of the revised Ability for Basic Movement Scale (ABMS II) for chronic-phase hospitalized patients, whether admitted owing to illness or injury.

Methods

A retrospective cohort of 176 consecutively admitted chronic care inpatients who underwent rehabilitation therapy between April 2021 and March 2023 was analyzed. Patients who died during admission, who were discharged within one month, or who had insufficient clinical data were excluded. Information on sex, age, residence before admission, and underlying diseases requiring physical rehabilitation therapy was collected. Daily living abilities were measured using the Functional Independence Measure (FIM) and Barthel Index (BI) scores during admission. Basic movement abilities were evaluated by two physical therapists using the ABMS II. The inter-rater reliability of ABMS II scores between therapists was analyzed using the weighted kappa coefficient. Spearman’s rank correlation coefficient was used to evaluate the validity of ABMS II and its correlation with FIM/BI scores. This retrospective study was approved by the Ethics Committee of Hikari Hospital (Approval No. HH‑2024‑01).

Results and discussion

The cohort had a mean age of 84.8 ± 7.3 years, 56% were female, and baseline mean (SD) scores were 18.6 ± 7.6 for ABMS II, 53.8 ± 22.9 for FIM‑total, and 35.0 ± 26.7 for BI. The ABMS II showed excellent agreement (weighted kappa = 0.996; 95% confidence interval: 0.993-0.999) and strong positive correlations (0.743-0.819) with total FIM/BI scores.

Conclusions

The ABMS Ⅱ is useful for evaluating basic movement abilities in patients with chronic illnesses.

## Introduction

Rehabilitation is crucial for maintaining and improving physical function and activities of daily living (ADLs) in older patients. In Japan, older patients hospitalized for illness or injury receive rehabilitation during the acute (the immediate phase after onset, which takes place in an acute care hospital), recovery (the phase of functional improvement, which takes place in a rehabilitation ward), and chronic (the long-term care phase, which takes place in a community-based or long-term care hospital) phases. Patients who cannot be discharged from acute or recovery rehabilitation hospitals continue rehabilitation to maintain and improve ADLs [1]. In this context, chronic care wards in Japan provide long-term medical and rehabilitation support for patients who are medically stable but still require ongoing assistance with ADLs. This level of care is broadly comparable to that provided by skilled nursing facilities in the United States.

Assessing basic movement abilities and ADLs is essential when providing rehabilitation for patients in the chronic phase of illness. The Functional Independence Measure (FIM) and Barthel Index (BI) scores are widely used to assess ADLs [2-6] and have demonstrated reliability and validity. However, these scales are qualitative and do not objectively measure patients’ basic movement abilities. In contrast, tools such as the Rivermead Mobility Index [7], Stroke Rehabilitation Assessment of Movement (STREAM) [8], Berg Balance Scale [9,10], and Trunk Control Test [11,12] provide more quantitative assessments; however, they often require professional expertise and specialized equipment.

To address the need for a simple and practical tool, the original Ability for Basic Movement Scale (ABMS) [13] was developed in Japan. It was designed to be used at the bedside by a wide range of healthcare professionals without requiring extensive training or special equipment. The ABMS allows for the evaluation of fundamental movements that are critical for daily functioning but are not adequately captured by standard ADL scales.

The revised version, the Ability for Basic Movement Scale II (ABMS II), has improved upon the original by refining its scoring system and enhancing its objectivity. Unlike other tools, such as the Rivermead Mobility Index or the STREAM score, which often target specific conditions like strokes or rely on the use of equipment, the ABMS II can be applied across a wide variety of conditions and care settings. It quantitatively assesses five essential movements: turning from a supine position, sitting up, sitting balance, standing up, and standing balance. This makes it particularly useful in long-term and community-based care environments. Its simplicity, reproducibility, and applicability in both acute and chronic phases of illness set it apart from more specialized or resource-intensive tools.

The ABMS II was developed by Tanaka et al. [14] as a revised version of the original ABMS. They demonstrated that, in patients with acute stroke, ABMS II scores could quantitatively predict factors associated with ADLs at discharge. Subsequent studies have shown that the ABMS II is also effective in predicting independent ambulation and ADLs at discharge in patients recovering from strokes or femoral fractures [15-18]. However, its reliability and validity have not yet been fully evaluated in patients in the chronic phase of illness. To our knowledge, no prior study has examined these measurement properties specifically in chronically hospitalized patients.

When older patients are discharged from chronic-phase hospitals and continue rehabilitation at home, in care facilities, or other clinical settings, sharing information on their basic movement abilities becomes especially important. A tool like the ABMS II, which is simple, convenient, and objective, allows healthcare professionals, such as visiting rehabilitation staff, care managers, and home medical teams, to effectively communicate patient function, optimize rehabilitation planning, support independence, and improve quality of life. Therefore, this study aimed to assess the utility of the ABMS II in evaluating basic movement abilities in patients during the chronic phase of illness.

## Materials and methods

Study design

This retrospective study investigated hospitalized patients who received rehabilitation therapy in the chronic care ward of Hikari Hospital between April 2021 and March 2023. Patients who died during hospitalization, those discharged within one month, or those who had insufficient clinical data were excluded. This study was approved by the Ethics Committee of the Hikari Hospital (January 31, 2024). The Committee waived the requirement for informed consent from patients and their families owing to the study’s retrospective nature.

Data collection

The following baseline patient characteristics were collected from medical records: sex, age, underlying diseases requiring rehabilitation therapy, disease classification, and patient residence before admission (home, facilities for older adults, or hospital admission). Patients’ ADLs were measured using the revised ABMS Ⅱ [14], FIM [4], and BI [2] scores.

Two physical therapists (PT1, eight years of experience; PT2, 15 years of experience) independently abstracted basic movement data and retrospectively converted them to ABMS II scores, blinded to each other’s ratings. Prior to abstraction, both raters jointly reviewed the ABMS II scoring manual, developed a shared template containing operational definitions for each level (1-6), and pilot-scored the first five charts. Full agreement was achieved, and the same template was then applied to all remaining records; any subsequent ambiguities were resolved by discussion.

Evaluation using the ABMS II [14] encompasses five basic movements: turning from a supine position, sitting up, remaining seated, standing up, and remaining standing. Each item was scored from 1 to 6 based on the patient’s ability. The grades of ability were as follows: 1 = prohibited from moving (because of a medical problem, such as unstable vital signs or complications); 2 = totally dependent; 3 = partially dependent; 4 = supervised (movement requiring verbal cues or gestures without physical contact); 5 = independent in a special environment (by holding a handrail or edge of the bed); and 6 = completely independent (without holding a handrail or edge of the bed). The minimum and maximum ABMS II scores were 5 and 30, respectively.

Assessment using the FIM [4] evaluates ADL performance in patients. It consists of two sections: a motor subscore (FIM-motor) and a cognitive subscore (FIM-cognitive). The motor items include eating, grooming, bathing, upper and lower body dressing, toileting, bladder and bowel management, bed/chair/wheelchair transfer, toilet transfer, bathtub/shower transfer, walking/wheelchair locomotion, and stair locomotion. The cognitive items include comprehension, expression, social interaction, problem-solving, and memory. Each item is scored on a scale of 1 to 7. The FIM-motor, FIM-cognitive, and total FIM score ranges include 13-91, 5-35, and 18-126, respectively.

Assessment using the BI [2] evaluates patients’ ADL capability using 10 subdivision items: feeding, bathing, grooming, dressing, bowel and bladder control, toilet use, transfer, mobility, and stair use. Each item is scored 0, 5, 10, or 15. The minimum and maximum BI scores are 0 and 100, respectively.

The revised ABMS II, FIM, and BI are all open-access tools published in peer-reviewed journals and are freely available for clinical and academic use [2,4,14]. Their broad application and validation across various populations support their use in this study without requiring special licensing or permission.

Statistical analyses

Normality of all continuous variables (ABMS II, FIM‑motor, FIM‑cognitive, FIM‑total, and BI at both evaluations) was assessed with the Shapiro-Wilk test, and none showed a normal distribution (all p < 0.05). Consequently, non‑parametric procedures were adopted. The inter‑rater reliability of ABMS II scores between physical therapists (PTs) was analyzed with the weighted kappa coefficient. Within‑patient changes between the initial and final evaluations were tested with the Wilcoxon signed‑rank test. Associations between ABMS II and FIM/BI scores were examined using Spearman’s rank correlation coefficient. Statistical significance was set at p < 0.05. The weighted kappa coefficients ranged from 0 to 1; values near 1 indicate higher agreement. Grading criteria were as follows: no agreement < 0; slight = 0.00-0.20; fair = 0.21-0.40; moderate = 0.41-0.60; substantial = 0.61-0.80; and almost perfect = 0.81-1.00 [19]. Spearman’s rho was interpreted as very high (0.90-1.00), high (0.70-0.89), moderate (0.50-0.69), low (0.30-0.49), and negligible (0.00-0.29) [20]. All analyses were performed with SPSS Statistics version 24 (IBM Corp., Armonk, NY). No formal a priori sample size calculation was conducted; however, a post‑hoc evaluation based on Donner & Eliasziw [21] indicated that the sample (n = 176) affords 80% power to detect a κ of 0.7 versus a null value of 0.4.

## Results

Patient characteristics

A total of 176 patients (78 men and 98 women) were included in this study, with a mean age (± standard deviation) of 84.8 (± 7.3) years. A total of 36, 64, and 76 patients were prescribed physical therapy for cerebrovascular disease (e.g., stroke, cerebral hemorrhage, and subarachnoid hemorrhage), immobility-related conditions (e.g., disuse syndrome due to prolonged bed rest or hospitalization), and motor disorders (e.g., osteoarthritis, fractures, and spinal disorders), respectively, based on classifications used in the Japanese rehabilitation reimbursement system. Bone fractures were the most common condition (22.7%), followed by musculoskeletal disorders (22.1%) and strokes (9.0%). More than half of the patients (56.8%) were transferred to another hospital (Table 1).

**Table 1:** Baseline patient characteristics (n = 176). n: number; %: percentage; SD: standard deviation. Age is presented as mean ± standard deviation (range). Other variables are presented as n (%).

		n	%	Mean ± SD (range)
Sex (Male:Female)		78:98	44.3:55.7	
Age (years)				84.9 ± 7.4 (65-102)
Physical therapy (%)				
	For cerebrovascular disease	36	20.4	
	For immobility	64	36.3	
	For motor disorder	76	43.1	
Disease classification (%)				
	Infectious disease	15	8.5	
	Cancer	7	3.9	
	Metabolic disease	3	1.7	
	Psychiatric disorder	1	0.5	
	Neurological disease	12	6.8	
	Circulatory system disease	11	6.2	
	Stroke	16	9	
	Respiratory disease	18	10.2	
	Digestive system disease	7	3.9	
	Dermatosis	1	0.5	
	Musculoskeletal disorder	39	22.1	
	Renal urological disease	2	1.1	
	Injury	4	2.2	
	Bone fractures	40	22.7	
Place of residence				
	Home	64	36.3	
	Facilities for older adults	12	6.8	
	Hospital	100	56.8	

The ABMS II scores at the initial and final evaluations were as follows: PT1 = 18.60 ± 7.60 (minimum-maximum = 5-30) and 20.09 ± 7.42 (6-30), respectively; PT2 = 18.51 ± 7.53 (5-30) and 20.03 ± 7.38 (6-30).

The results of the ABMS II, FIM, and BI scores at the initial and final evaluations are summarized in Table 2.

**Table 2:** Result of clinical data (ABMS II/FIM/BI). Mean ± standard deviation and median (interquartile range). ABMS II: revised Ability for Basic Movement Scale; FIM: Functional Independence Measure; BI: Barthel Index; PT: physical therapist; Initial: within one week of rehabilitation therapy initiation; Final: within two weeks of discharge or transfer to the treatment ward from the community-based care ward.

		Initial			Final		p-value
		Mean ± SD	Median (IQR)		Mean ± SD	Median (IQR)	
ABMS Ⅱ	PT 1	18.60 ± 7.60	20 (11.25–26)		20.09 ± 7.42	21.5 (14.25–26)	<0.001
	PT 2	18.51 ± 7.53	20 (12–26)		20.03 ± 7.38	21 (15–26)	<0.001
FIM	Motor	33.45 ± 16.37	32.5 (19–46.75)		36.18 ± 18.53	33 (21–51)	<0.001
	Cognitive	20.64 ± 8.20	21 (14–27)		20.42 ± 8.48	20 (14–27)	0.855
	Total	53.84 ± 22.85	52 (36–71.75)		56.59 ± 25.53	55 (35.5–77)	<0.001
BI		35.03 ± 26.73	35 (10–60)		39.37 ± 29.39	40 (15–65)	<0.001

Baseline score distributions were left‑skewed with minimal ceiling effects: only 3% of patients scored ≥ 28 on the ABMS II, and 5% scored ≥ 90 on the BI. With the exception of the FIM‑cognitive domain (p = 0.855), all indices showed statistically significant improvements between admission and discharge (p < 0.001).

Weighted kappa and Spearman’s rank correlation coefficients

The weighted kappa coefficients for the initial and final evaluations were 0.996 (p < 0.001, 95% confidence interval (CI): 0.993-0.998) and 0.996 (p < 0.001, 95% CI: 0.993-0.999), respectively (Table 3).

**Table 3:** Weighted kappa coefficients for the initial and final evaluations. ABMS II: revised Ability for Basic Movement Scale; CI: confidence interval; Initial: within one week of rehabilitation therapy initiation; Final: within two weeks of discharge or transfer to the treatment ward from the community-based care ward.

ABMS Ⅱ	Weighted kappa coefficient		95% CI				p-value
Initial	0.996		0.993	–	0.998		<0.001
Final	0.996		0.993	–	0.999		<0.001

All associations were positive. ABMS II exhibited strong correlations with both total FIM and BI scores (r = 0.743-0.885) and with the FIM‑motor domain (r = 0.827-0.867), while its correlation with the FIM‑cognitive domain was moderate (r = 0.438-0.651) (Table 4).

**Table 4:** Spearman’s rank correlation coefficient between the ABMS II and FIM/BI scores. All correlations were positive and statistically significant at p < 0.001 unless otherwise noted. 95 % CI calculated with Fisher’s r-to‑z transformation. ABMS II: revised Ability for Basic Movement Scale; FIM: Functional Independence Measure; BI: Barthel Index; PT: physical therapist; Initial: within one week of initiating rehabilitation therapy; Final: within two weeks of discharge or transfer to the treatment ward from the community-based care ward.

	ABMS II						
	Initial				Final		
	PT1		PT2		PT1		PT2
	r (95 % CI)		r (95 % CI)		r (95 % CI)		r (95 % CI)
FIM							
Motor	0.827 (0.772–0.870)		0.828 (0.773–0.870)		0.867 (0.823–0.900)		0.865 (0.821–0.899)
Cognitive	0.438 (0.306–0.553)		0.443 (0.312–0.558)		0.651 (0.554–0.731)		0.649 (0.551–0.729)
Total	0.743 (0.666–0.804)		0.745 (0.669–0.806)		0.846 (0.797–0.885)		0.844 (0.793–0.883)
BI	0.818 (0.761–0.863)		0.819 (0.761–0.863)		0.885 (0.808–0.891)		0.855 (0.808–0.891)

## Discussion

This study provides convergent evidence that the revised Ability for Basic Movement Scale II (ABMS II) is both reliable and valid in a chronic‑phase inpatient cohort. Inter‑rater agreement was excellent, with identical weighted kappa coefficients of 0.996 for the initial and final assessments, categorised as “almost perfect” reliability. Concurrently, ABMS II demonstrated strong positive correlations with established functional measures: r = 0.743-0.885 for total FIM and BI scores and r = 0.827-0.867 for the FIM‑motor domain. Together, these metrics confirm that ABMS II can be applied with confidence to quantify basic movement abilities in older adults receiving long‑term hospital rehabilitation.

Mean scores showed statistically significant but quantitatively small improvements from baseline to discharge (ABMS II +1.5 points; FIM‑total +2.8 points; BI +4.3 points; all p < 0.001). Published minimal clinically important differences (MCIDs) for comparable chronic care cohorts are ≈17 points for the FIM‑motor domain and ≈10 points for the BI [22,23]. An MCID for the ABMS II has not yet been established; however, applying a distribution‑based criterion of 0.2 SD yields 1.5 points in our sample, which matches the observed change. Thus, the ABMS II improvement meets a small‑effect threshold, whereas the FIM and BI gains, although statistically significant, likely fall below commonly accepted benchmarks for clinical relevance. Determining the responsiveness and definitive MCID of the ABMS II was outside the scope of this study and warrants investigation in future prospective work.

To our knowledge, this is the first study to quantify inter-rater reliability of the ABMS II in a chronic-phase inpatient cohort, and the almost-perfect κ = 0.996 confirms that the scale remains highly reproducible even when scored retrospectively from routine clinical notes.

Our study also showed a strong correlation between ABMS II and FIM/BI scores at both the initial and final evaluations (0.743-0.819), which has not been described in previous studies. Tanaka et al. [14] reported a strong correlation between ABMS Ⅱ and BI scores in 71 patients with acute strokes (age = 71.2 ± 13.5 years; cerebral infarctions: 38, cerebral hemorrhages: 33) four weeks after onset. The disease phase of the patients in their study differed from that in ours. Our results support the correlation between ABMS Ⅱ scores and assessment tools for evaluating patient ADLs, such as the FIM and BI scores.

The ABMS II scores correlated more strongly with the FIM-motor scores (r = 0.827-0.867) than with FIM-cognitive scores (r = 0.438-0.651) in this study, indicating that the ABMS II better reflects motor function than cognitive function. Takei et al. [24] reported that patients’ transfer ability (moving between a bed and a wheelchair) may be influenced not only by lower-limb paralysis and cognitive dysfunction but also by their ability to stand and sit. Our results are consistent with the findings of a previous study [24], suggesting that the results of the ABMS II were more relevant for assessing FIM-motor than FIM-cognitive in patients hospitalized in the chronic phase of illness. This may be because the ABMS II involves only motor function evaluation and does not include evaluation of cognitive function, resulting in a weaker correlation with FIM-cognitive scores.

Although ABMS II was designed to capture elemental movement capacities that precede complex ADLs, its moderate to strong positive associations with both FIM and BI suggest sound convergent validity. Perfect agreement with these composite ADL indices is neither expected nor desirable; rather, the partial correlation indicates that ABMS II provides complementary information on the motor prerequisites of functional independence. Future research should extend validation to criterion measures that reflect raw performance, such as gait speed, chair rise time, or wearable sensor metrics, to delineate how changes in fundamental movement ability translate into day-to-day functional gains.

In this study of 176 patients hospitalized in the chronic phase of illness, the ABMS Ⅱ demonstrated high reliability between PTs and a strong correlation with FIM/BI scores at both the initial and final evaluations. These findings suggest that the ABMS Ⅱ effectively evaluates basic movement ability in patients hospitalized in the chronic phase of illness. As this is the first report on the application of the ABMS Ⅱ in the chronic phase of illness, further studies utilizing the ABMS Ⅱ are needed to confirm these findings.

This study had some limitations. First, this retrospective study was conducted at a single chronic care hospital; future multicenter studies should include patients in other phases of disease progression. Second, patient anamnesis was not investigated, leaving its impact on patients’ basic movement abilities unclear; future studies should evaluate this. Additionally, two PTs retrospectively converted patients’ basic movement abilities to ABMS Ⅱ scores based on rehabilitation records; thus, these abilities were not directly examined in this study. To evaluate reliability, a prospective study should directly evaluate these abilities through prospective or external validation. Fourth, analyses were not stratified by primary diagnosis, and potential variation in the performance and validity of ABMS II across disease groups (e.g., cerebrovascular, musculoskeletal, and disuse) remains to be elucidated.

## Conclusions

In conclusion, this study establishes that the ABMS II is both highly reliable and strongly valid, with robust correlations to FIM and BI scores, in older adults hospitalized during the chronic phase of illness. This is the first investigation to confirm the scale’s psychometric properties in a heterogeneous chronic care inpatient cohort, thereby closing the evidence gap left by prior research restricted to acute or post‑acute populations. Beyond its psychometric soundness, the scale’s brevity and bedside feasibility give it immediate clinical utility: (i) it furnishes an objective metric that can be shared across disciplines, thereby streamlining hand‑offs between physicians, nurses, physical therapists, and care‑managers; (ii) its quantitative scores aid discharge planning by helping clinicians differentiate patients who may safely return home from those requiring facility‑based rehabilitation; and (iii) serial ABMS II assessments provide a concise dashboard for tracking functional trajectories during long‑term care and community follow‑up. These advantages underscore the scale’s potential to enhance decision‑making and interdisciplinary communication in chronic‑care settings. Future prospective multicenter investigations should not only corroborate these results but also establish clinically useful cut‑off values and minimally clinically important differences for the ABMS II, as well as clarify its responsiveness to functional change over time.
